# Precision prevention of monkeypox

**DOI:** 10.1093/pcmedi/pbac026

**Published:** 2022-10-20

**Authors:** Kang An, Zhenmi Liu, Furong Qu, Zhenmei An

**Affiliations:** General Practice Ward/International Medical Center Ward, General Practice Medical Center, National Clinical Research Center for Geriatrics, West China Hospital, Sichuan University, Chengdu 610041, China; West China School of Public Health and West China Fourth Hospital, Sichuan University, Chengdu 610041, China; General Practice Ward/International Medical Center Ward, General Practice Medical Center, National Clinical Research Center for Geriatrics, West China Hospital, Sichuan University, Chengdu 610041, China; Department of Endocrinology and Metabolism, West China Hospital, Sichuan University, Chengdu 610041, China; Department of Endocrinology and Metabolism, West China Hospital, Sichuan University, Chengdu 610041, China; Department of Laboratory Medicine, West China Hospital, Sichuan University, Chengdu 610041, China

Dear Editor,

Recently, the World Health Organization (WHO) declared monkeypox as a Public Health Emergency of International Concern (PHEIC), another one after the last PHEIC of COVID-19 announced in early 2020.^1^ Unlike the new SARS-CoV-2 virus that has multiple variants, monkeypox virus is a DNA virus first identified in 1958 with no evidence of significant mutation of its sequence.^2^ Currently, the cause of the monkeypox outbreak remains unclear and further investigation is urgently needed. Like other transmitting diseases, large number of asymptomatic cases may exist,^3^ with the exact data unclear in the current outbreak. Most recent studies reported close contact with rash lesions or body fluid of infected individuals, including sexual intercourse, kissing, hugging, massaging and cuddling.^4,5^

In the era of precision medicine, precision prevention has changed the traditional one-size-fits-all prevention strategies by developing more tailored and effective strategies for different risk populations, notably in identification, assessment, risk stratification, and precaution. Since monkeypox poses significant pressure to public health and challenges healthcare resources, its precision prevention, for countries either with or without reported cases, is crucial for controlling the pandemic with low cost. The world pandemics of COVID-19 triggered the improvement of the virus control for travel-associated spread across countries. Similarly, properly planned public health policies, if executed adequately in time, may have good chances to stop ongoing spread of monkeypox virus. Though monkeypox has lower transmissibility than COVID-19, the experiences from the latter appeal governments and public health systems to take action as soon as possible before the virus gets out of control. Precision identification of people at risk and precision prevention strategies can facilitate the coordination of disease control and cut the impact of the virus before it affects wider population. The WHO has provided temporary recommendations that categorize the risk of countries into four tiers, and for each tier there are recommendations for actions.^1^ Group 1 countries are those with no past cases or newly detected case within 21 days. Stopping imported cases is the most critical issue and the public awareness of the disease is essential. Group 2 countries are those with imported cases and may have human-to-human transmission. For such countries, transparency of case report and precision prevention for people at risk are of the top priority. Smallpox vaccination could be necessary too. Group 3 countries are those with known or suspected animal-to-human transmission, meaning monkeypox could be hardly eliminated and long-term disease control measures are required. Group 4 countries have manufacturing capacity for medical countermeasures. These countries have responsibility to work with the WHO to ensure that necessary public health supplies are available to the most needed countries.

For countries at high risks, we propose that the public health systems identify people as different risk tiers (Table 1) for precision prevention of the disease: people unlikely to exposure (Tier 1), people with chance to exposure (Tier 2), people exposed to the virus (Tier 3), and people with virus infected (Tier 4). Similarly, the management objectives for each tier of people as well as the time and quality of management actions decide the effectiveness of the disease control. Unlike COVID-19, people unlikely to monkeypox exposure do not need specific precaution but need to be aware of the basic knowledge of the disease. Precision prevention does not only benefit them when they encounter monkeypox infection risks, but also prevent disease stigma during monkeypox outbreak. For people with chance to exposure, smallpox vaccination provides cross protection against monkeypox and could be necessary. For people exposed to the disease, clinical assessment as early as possible is needed with standard precaution. For confirmed cases, routine supportive care can reduce discomfort and help them recover early. To be noted, the skin lesions, especially those on the face and other exposing areas, can leave an identification for the infected experience and should be controlled as a priority.

**Table 1:** Risk tiers for people and corresponding goals and actions.

Risk tiers	Description	Objective	Action
1	People unlikely to exposure	To understand the disease correctly	Receiving public health education
2	People with chance to exposure	To reduce exposure and improve immunity	Receiving public health education and vaccination
3	People exposed to the virus	To assess the infection and to reduce transmission	Early assessment and precaution
4	People with virus infected	To recover early and prevent sequela (eg. scars) and death	Precaution and medical care

Western countries including European Union countries, the United Kingdom, and the United States (US) have initiated precision prevention of monkeypox cases.^4,6^ The US highlights the importance of health communication and works with state and local partners to develop and provide tailored harm reduction messages to diverse communities.^6^ For example, the advocation of fewer one-time sex partner may protect people at risk.^7^

To be noted, precision prevention has its drawbacks if used inappropriately. The disease-related stigma is a common and substantial obstacle to the control of transmitting disease.^6^ People who are at risks of Tier 2 to 4 may face social and psychological attacks if personal information is improperly disclosed. Privacy protection is thus critical during application of precision prevention. It also calls for early public education that guides the public to correctly understand the disease and treat the infected persons fairly.

Although many currently reported cases were men who have sex with men (MSM) and people with HIV infection, the causal risk factors for monkeypox remain uncertain.^4^ This population overlapping could be multifactorial and the potential founder effects should be noted. Hence, with more communities infected by the virus, more transmitting routes may be identified. Another knowledge limitation comes from the neglect of asymptomatic cases, as the current case series described only symptomatic ones.^3^ Though immunosuppression associated with HIV infection magnifies the severity and transmission of the disease, HIV infection is not a prerequisite of monkeypox infection. The interactions between immunosuppression, low vaccination, and detection rate of infected individuals are thus complicated and demand further investigations. Identifying infected individuals and those at high risk warrants well-designed controlled studies to confirm the risk factors that allow the risk stratification.

For infected people, treatments are employed to relieve symptoms and prevent complications due to the self-limiting nature of monkeypox. Certain patients, for example the immunosuppressed population, may benefit from antiviral therapies, including tecovirimat and brincidofovir. With less adverse events compared to brincidofovir,^8^ tecovirimat is now the first-line antiviral drug for monkeypox approved by the European Medicines Agency. Clinicians should stay alert of secondary bacterial infection which can be treated effectively with antibiotics. In light of the safety data and unknown risks for long-term medication use, therapeutic decisions should be made jointly by clinicians and public health professionals. With credible evidence, drug regulatory agencies should accelerate the assessment and approval of products under development, facilitate clinical trials and support the fast-track approval of medicines such as vaccines and antivirals through innovative approaches and regulatory flexibility. It should also be noted that clinicians should not prescribe unapproved drugs to patients unless in very urgent and severe cases.

Key pathway to prevention against monkeypox is vaccination. WHO recommends urgent use of vaccination after exposing to infected individuals or their contacts, including healthcare worker. The ring vaccination strategy has been implemented by India, the USA, and several European countries.^9^ This strategy relies on precision prevention to rapidly identify and contact tracing. With increased availability of vaccination, people at risk could apply before exposure or when the exposure is unclear.^9^ ACAM2000 (live) and JYNNEOS (live, nonreplicating) are currently available in some countries.^10^ For post-exposure prophylaxis in highly immunocompromised patients, there is no definitive evidence for use of vaccinia immune globulin. Addressing inequities in vaccine availability and distribution is an urgent global priority, especially in parts of Central and West Africa. Using a smaller dose with intradermal injection could extend vaccine supply. Further evaluation is needed to assess efficacy and safety.

Viruses know no borders and all humanity should stand together. Precision public health and medicine may reform the epidemic and pandemic preparedness and response. The precision responses to monkeypox threats should be built on not just accurate and rapid disease control and treatment, but also public health equity and fairness.

## References

[1] World Health Organization. Second meeting of the International Health Regulations (2005) (IHR) Emergency Committee regarding the multi-country outbreak of monkeypox. Available from: https://www.who.int/news/item/23-07-2022-second-meeting-of-the-international-health-regulations-(2005)-(ihr)-emergency-committee-regarding-the-multi-country-outbreak-of-monkeypox (20 September 2022, date last accessed).

[2] Damon IK. Status of human monkeypox: clinical disease, epidemiology and research. Vaccine. 2011;29(Suppl 4):D54–9.. doi:10.1016/j.vaccine.2011.04.014. 22185831 10.1016/j.vaccine.2011.04.014

[3] De Baetselier I , Van Dijck C, Kenyon C et al. Retrospective detection of asymptomatic monkeypox virus infections among male sexual health clinic attendees in Belgium. Nat Med. 2022. doi:10.1038/s41591-022-02004-w. 10.1038/s41591-022-02004-w PMC9671802 35961373

[4] Tarín-Vicente EJ , Alemany A, Agud-Dios M et al. Clinical presentation and virological assessment of confirmed human monkeypox virus cases in Spain: a prospective observational cohort study. Lancet North Am Ed. 2022;400(10353):661–9.. doi:10.1016/S0140-6736(22)01436-2. 10.1016/S0140-6736(22)01436-2 PMC9533900 35952705

[5] Liu D , Chi Y, Song P et al. Risk factors, clinical manifestation, precaution, and management of monkeypox. J Evid Based Med. 2022;15(3):183–6.. doi:10.1111/jebm.12490. 35996882 10.1111/jebm.12490

[6] Delaney KP , Sanchez T, Hannah M et al. Strategies adopted by gay, bisexual, and other men who have sex with men to prevent monkeypox virus transmission—United States, August 2022. MMWR Morb Mortal Wkly Rep. 2022;71(35):1126–30.. doi:10.15585/mmwr.mm7135e1. 36048582 10.15585/mmwr.mm7135e1 PMC9472779

[7] Spicknall IH , Pollock ED, Clay PA et al. Modeling the impact of sexual networks in the transmission of monkeypox virus among gay, bisexual, and other men who have sex with men—United States, 2022. MMWR Morb Mortal Wkly Rep. 2022;71(35):1131–5.. doi:10.15585/mmwr.mm7135e2. 36048619 10.15585/mmwr.mm7135e2 PMC9472773

[8] Adler H , Gould S, Hine P et al. Clinical features and management of human monkeypox: a retrospective observational study in the UK. Lancet Infect Dis. 2022;22(8):1153–62.. doi:10.1016/S1473-3099(22)00228-6. 35623380 10.1016/S1473-3099(22)00228-6 PMC9300470

[9] Choudhary OP , Priyanka, Fahrni ML, et al. Ring vaccination for monkeypox containment: Strategic implementation and challenges. Int J Surg. 2022;105:106873. doi:10.1016/j.ijsu.2022.106873. 36055631 10.1016/j.ijsu.2022.106873 PMC9424115

[10] Vaughan A , Aarons E, Astbury J et al. Human-to-human transmission of monkeypox virus, United Kingdom, October 2018. Emerg Infect Dis. 2020;26(4):782–5.. doi:10.3201/eid2604.191164. 32023204 10.3201/eid2604.191164 PMC7101111

